# Identifying Images of Dead Chickens with a Chicken Removal System Integrated with a Deep Learning Algorithm

**DOI:** 10.3390/s21113579

**Published:** 2021-05-21

**Authors:** Hung-Wei Liu, Chia-Hung Chen, Yao-Chuan Tsai, Kuang-Wen Hsieh, Hao-Ting Lin

**Affiliations:** Department of Bio-Industrial Mechatronics Engineering, National Chung Hsing University, Taichung 402, Taiwan; s106040023@mail.nchu.edu.tw (H.-W.L.); s108040002@mail.nchu.edu.tw (C.-H.C.); yctsaii@dragon.nchu.edu.tw (Y.-C.T.); cwshieh@dragon.nchu.edu.tw (K.-W.H.)

**Keywords:** broiler, dead chicken, poultry house, removal system, YOLO v4

## Abstract

The chicken industry, in which broiler chickens are bred, is the largest poultry industry in Taiwan. In a traditional poultry house, breeders must usually observe the health of the broilers in person on the basis of their breeding experience at regular times every day. When a breeder finds unhealthy broilers, they are removed manually from the poultry house to prevent viruses from spreading in the poultry house. Therefore, in this study, we designed and constructed a novel small removal system for dead chickens for Taiwanese poultry houses. In the mechanical design, this system mainly contains walking, removal, and storage parts. It comprises robotic arms with a fixed end and sweep-in devices for sweeping dead chickens, a conveyor belt for transporting chickens, a storage cache for storing chickens, and a tracked vehicle. The designed system has dimensions of approximately 1.038 × 0.36 × 0.5 m^3^, and two dead chickens can be removed in a single operation. The walking speed of the chicken removal system is 3.3 cm/s. In order to enhance the automation and artificial intelligence in the poultry industry, the identification system was used in a novel small removal system. The conditions of the chickens in a poultry house can be monitored remotely by using a camera, and dead chickens can be identified through deep learning based on the YOLO v4 algorithm. The precision of the designed system reached 95.24% in this study, and dead chickens were successfully moved to the storage cache. Finally, the designed system can reduce the contact between humans and poultry to effectively improve the overall biological safety.

## 1. Introduction

Broilers are the major product of the Taiwanese poultry industry. Due to increased demands for chicken, the feeding size of the Taiwanese poultry industry has increased. The Taiwanese chicken population is also aging. Thus, poultry breeding management is crucial. Consequently, many businesses have switched from traditional extensive breeding methods to specialized breeding methods that involve using environmental sensors and robots. These specialized methods can be combined with autonomous mobile platforms to enhance the automation and artificial intelligence in the poultry industry, thus, promoting its development.

By using intelligent poultry systems, a breeder can obtain information on the environment in a poultry house, including the temperature, humidity, CO_2_ concentration, wind speed, and ammonia content, on a big data platform through a remote method. The installation of technological devices in a poultry house enables the efficient and early detection of potentially abnormal environmental situations to improve poultry welfare and reduce the environmental impact of the industry [1,2].

Breeders must regularly perform repetitive and dirty tasks, including stimulating poultry activity, observing chicken health, turning the litter to prevent the breeding of germs, and removing dead chickens, in poultry houses every day. However, the entry of a breeder into a poultry house can result in the spread of germs, which increases the possibility of cross-infection between chickens and humans. To solve this problem, many researchers have installed environmental sensors and various functional mechanisms on small robots. Small robots that can perform tasks in poultry houses have been developed for many years.

The French company Octopus Robots [3] is exclusively devoted to the development of robots for poultry houses. The company’s robots are called Octopus Poultry Safe robots. These robots ventilate litters by turning them to prevent germ reproduction. They are equipped with a disinfection system and facilitate the penetration of disinfectant into the litters. TIBOT Technologies [4], a French company, consisting of breeders, entrepreneurs, and engineers, designed a robot called Spoutnic NAV that can turn and aerate litters. The robot can move randomly in a barn such that the hens in the barn are forced to move continuously and prevented from laying eggs randomly.

At the end of the breeding cycle, poultry harvesting by the breeder requires considerable labor over a few days. To solve this problem, large robots that can remove chickens from a poultry house have been developed for the poultry industry. These robots enable labor to be replaced with machines to relieve the breeder’s burden and increase the harvesting efficiency. However, chicken harvesting systems can harvest only adult chickens. CMC Industries (https://www.cmcindustries.com, (accessed on 19 May 2021)) developed a harvesting system called Apollo Generation 2. In this system, the two wings of the front collection head, which are made of conveyor belts, can be opened. The Danish company JTT Conveying A/S (https://www.jtt.dk/, (accessed on 19 May 2021)) developed the Chicken Cat system to harvest broilers.

The collector in this system consists of three barrel-like drums that rotate against each another. The system can harvest 7000 chickens per hour. Currently, dead chickens in Taiwanese poultry houses are removed manually by breeders who patrol the houses daily to judge the health of the chickens on the basis of their breeding experience. The development of a robot that can autonomously remove dead chickens through object detection in poultry houses would not only reduce the time and labor costs but also human–poultry contact to improve biological safety. Deep learning is widely adopted for object detection in various fields, such as medical care [5,6], autonomous driving [7], and chicken industry [8].

Finally, the rest of this paper is organized as follows. Related works are described in Section 2. The chicken removal system is depicted in Section 3. The object detection method and analysis are described in Section 4. The evaluation of the chicken removal performance is discussed in Section 5. Section 6 is the conclusions.

## 2. Related Works

In 2020, Ren et al. [9] reviewed the agricultural robotics research applicable to poultry production and divided agricultural robots into three categories according to their functions: monitors, harvesters, and both monitors and harvesters. They found that numerous challenges must be overcome to mechanize agricultural tasks in general and poultry production in particular. Thus, high efficiency can be achieved in the management of poultry houses through automated tasks. Many self-propelled vehicles have been developed to replace labor. These vehicles can overcome not only the problem of labor shortages but also the problem of fixed-point environmental sensors, which may not accurately represent an entire poultry house.

Furthermore, the stress generated in broilers by robots is not greater than that generated by humans. According to Parajuli et al. [10] and Usher et al. [11], appropriately operated robots do not cause more fear among broilers than humans do; thus, robots can be used to improve the poultry industry. In 2009, Murad et al. [12] developed a monitoring system based on a wireless sensor network (WSN) for poultry farms. Their system comprises Crossbow’s TelosB motes integrated with commercial sensors that can measure temperature and humidity. The data collected by the sensors are uploaded to an online database to enable managers to obtain information from the online monitoring solution provided by the system.

In 2015, Mirzaee-Ghaleh et al. [13] monitored and maintained four indoor climate parameters, namely the temperature, humidity, CO_2_ concentration, and NH_3_ concentration, by using three self-developed fuzzy logic controllers. Research on artificial intelligence has indicated that smart control techniques, such as fuzzy systems, can not only maintain an indoor climate within acceptable margins but also reduce the energy consumption [14]. In 2016, Zhang et al. [15] proposed a system based on a WSN for controlling the environmental parameters in buildings housing livestock.

This system allows a breeder to monitor the temperature, humidity, lighting level, CO_2_ concentration, NH_3_ concentration, and H_2_S concentration in buildings housing livestock in real time. In addition, the aforementioned method reduces labor costs and energy consumption. In 2020, Astill et al. [16] investigated areas of the poultry industry affected by smart sensor technologies. They also described how sensor technology is related to big data analytics and Internet of Things systems. This technology can increase the output of the poultry industry. Thus, the introduction of technology to the poultry industry can increase the output and convenience of this industry.

In 2018, Vroegindeweij et al. [17] evaluated the performance of PoultryBot, which is an autonomous mobile robotic platform for poultry houses. PoultryBot helps breeders to assess chicken status and housing environments as well as collect floor eggs manually. PoultryBot achieves the goals of navigation and egg collection through localization and object detection. In 2020, Chang et al. [18] proposed a smart mobile robot for poultry houses that can recognize eggs of two colors on free-range farms. The robot can also pick up and sort eggs without damaging them.

The small smart mobile robots that are currently used in poultry houses can complete most tasks that were previously performed manually. In 1996, Chung Hsing University [19] introduced catching machinery produced by the Dutch company Pluriot-Ronico to Taiwan chicken. The KVM-500 catching machine has a length, width, and height of approximately 7.8, 2.0, and 1.9 m, respectively. The machine can harvest 3000–5000 chickens per hour with a vacuum pump. In 2005, Nijdam et al. [20] compared manually and mechanically caught broilers in terms of certain factors. According to the field trial conducted in the study, the catching method did not affect the percentage of bruises or the meat quality.

In 2020, C.L. Chowdhary et al. [21] proposed hybrid techniques for image encryption and decryption. Experiments showed that Elliptic Curve Cryptography (ECC) and Hill Cipher (HC) presented good solutions for image encryption. In 2020, Mohammad R. Khosravi et al. [22] proposed the toolbox of ENVI for several single-band images along with multi-band polarimetric SAR (Pol-SAR) images. Experiments show that some classic filters are better in comparison to newer filters. In 2021, Adam [23] proposed the fault diagnosis of electric impact drills using thermal imaging, the feature extraction of thermal images using Binarized Common Areas of Image Difference (BCAoID).

The recognition results were 97.91–100%. Fault diagnosis based on thermal images can protect rotating machinery and engines. In our study, distances between the chicken removal system and the measurement equipment are a large issue. However, thermal cameras have distance constraints when measuring the target. To obtain an accurate temperature, the distance between the thermal camera and target should not be too far. Therefore, the visible light camera was adopted for the chicken removal system in this study. In 2009, Zhu et al. [24] adopted a Support Vector Machine (SVM) algorithm for automatic dead chicken detection in modern chicken farms.

The experiments showed that the detection accuracy was over 90%. In 2018, Jake et al. [25] reviewed the research on detecting and predicting emerging diseases in poultry with the implementation of new technologies and big data. Avian influenza virus was the focus. In 2015, Sadeghi et al. [26] proposed an intelligent procedure for the detection and classification of chickens infected by clostridium perfringens based on their vocalization. The results demonstrated the usefulness and effectiveness of intelligent methods for diagnosing diseases in chickens. In 2020, Toğaçar et al. [27] developed a COVID-19 detection method with deep learning models. In the preprocessing step, the data classes were restructured using the Fuzzy Color technique, and the images were structured with the original images were stacked.

In 2017, Versaci et al. [28] proposed adaptive image contrast enhancement by computing distances into a 4-dimensional fuzzy unit hypercube. In this study, the problem of contrast enhancement for gray level images is solved with a new fuzzy procedure. In 2021, Neethirajan, S. [29] proposed a real-time emotion recognition system based on YoloV3, and Faster YoloV4-based facial detection platform and an ensemble Convolutional Neural Network (RCNN) for measuring emotions in farm animals.

Compared with the spacious breeding environments in poultry houses in other countries, poultry houses in Taiwan have smaller breeding environments (a column height of approximately 5–10 m); thus, the chicken harvesting systems developed in foreign countries are unsuitable for Taiwanese poultry houses. In addition, a removal system for dead chickens has not yet been developed. Therefore, a novel chicken removal system that is suitable for Taiwanese poultry houses was developed in this study by referring to the design of existing chicken harvesting mechanisms and miniaturizing them. This system performs object identification by using the YOLO v4 deep learning algorithm to detect dead chickens in Taiwanese poultry houses to enhance the system efficiency as well as achieve automation and artificial intelligence application in poultry houses. The designed system can solve the problems associated with the manual removal of dead chickens.

## 3. Chicken Removal System

**Figure 1:**
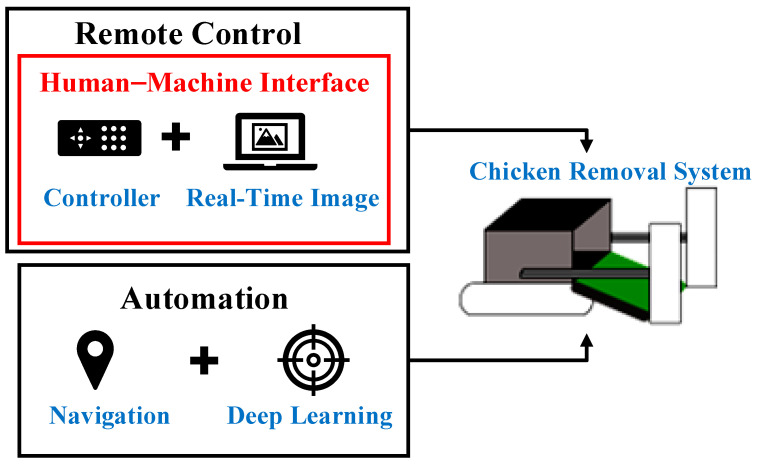
The concept of the small chicken removal system.

In this study, a small chicken removal system was developed for removing dead chickens from Taiwanese poultry houses. The concept of the small chicken removal system is displayed in Figure 1. Two modes are designed for the small chicken removal system. One is remote control, the designed system is connected to the user equipment through Wi-Fi to enable breeders to control the system via the human–machine interface from anywhere. Thus, breeders need not enter poultry houses to remove dead chickens manually. The other is the automation mode, the designed system can operate automatically without human intervention and perform tasks, such as navigation and deep learning for object detection.

### 3.1. Configuration of the Removal System

**Figure 2:**
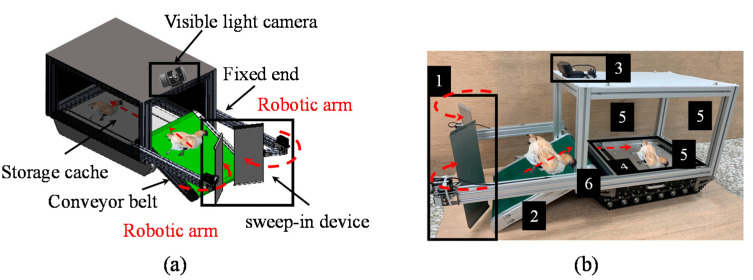
Design of the small chicken removal system: (**a**) schematic and (**b**) experimental setup. (1) Two stainless steel plates for rotational movement, (2) conveyor belt for single-axis movement, (3) visible light camera for capturing images, (4) storage cache for storing dead chickens, (5) three transparent acrylic sheets for the enclosed space, and (6) tracked vehicle.

The designed system is illustrated in Figure 2. This system is equipped with robotic arms, a conveyor belt, and a storage cache to remove dead chickens. The system can be operated in two modes: remote control and automatic. In the remote control mode, the breeder can click buttons on the human–machine interface to control the walking and removal systems. In addition, a visible light camera—a noncontact visible sensor—attached to the top of the storage cache is installed in the small chicken removal system. This camera can instantly transmit captured images to the user equipment. The angle of the visible sight is 78 degrees in the horizontal plane. Therefore, dead chickens should be in front of the removal system in this area.

The removal system is switched on when the breeder clicks the “Rotate” button for the conveyor belt and the “Open” button for the robotic arms after a dead chicken is detected. In automatic mode, the chicken removal system can automatically navigate and perform object detection in a poultry house. The design of the chicken removal system is based on the complex environment of Taiwanese poultry houses. The designed system has a length, width, and height of approximately 1.038, 0.36, and 0.5 m, respectively. The components, systems, and system models used in the designed system are presented in Table 1. The integration of the removal and walking systems enables the designed robot to walk in a poultry house and to remove dead chickens.

Most robots currently used in poultry houses use wheeled self-propelled vehicles. Such vehicles are used mainly because (1) wheeled vehicles are easy to disassemble and (2) relatively small amounts of feathers and litter stick to the tires of these vehicles. However, wheeled vehicles are less maneuverable than tracked vehicles are [30]. Furthermore, the poultry house floor is covered with litter, which may influence the walking performance.

Therefore, the high mobility of a tracked vehicle enables it to overcome bumpy landforms in a poultry house. In the walking system of the designed robot, the tracked vehicle (WT-500) has a length, width, and height of approximately 0.50, 0.30, and 0.15 m, respectively. A high-torque and low-speed (22 rpm) motor is used as a drive so that the tracked vehicle can walk stably in a poultry house. In addition, low-speed walking can reduce the disturbance in a poultry house and avoid raising dust.

The removal system mainly comprises three mechanisms: robotic arms, a conveyor belt, and a storage cache. The robotic arms have a fixed end and sweep-in device. The fixed end contains an aluminum extrusion (length = 350 mm) whose height and angle can be adjusted to adapt to the environments of different poultry houses and improve the feasibility of the designed system. A stainless steel plate (length of 300 mm and width of 110 mm) is used as the sweep-in device, and a servo motor is used to drive the rotation of this plate.

To sweep heavy chickens, a servo motor with a large torque (50 Kg-cm) is essential for driving the rotation of the stainless steel plate. Two robotic arms are present on either side in front of the entire designed system. The stainless steel plate on the left of the system rotates clockwise, and the stainless steel plate on the right rotates counterclockwise. When an object is between these two stainless steel plates and the conveyor belt, the sweeping action of the stainless steel plate used as the sweep-in device may be triggered.

When a dead chicken is swept in by the stainless steel plates, the conveyor belt transports the dead chicken to the storage cache. The conveyor belt (720 mm long and 259 mm wide) is made of polyvinyl chloride material, and its surface is rough to increase friction. To increase the adaptability of the field, the angle of the conveyor belt can be adjusted according to the height of the litter in different poultry houses. The storage cache is sealed with a transparent acrylic sheet to prevent any viruses in the dead chickens inside the cache from spreading to healthy chickens. The storage cache has a length, width, and height of approximately 0.45, 0.3, and 0.25 m, respectively. The internal dimensions of the storage cache were designed according to the size of the largest broiler in Taiwan. The storage cache can store two to three dead chickens.

### 3.2. Software and Control Architecture

**Figure 3:**
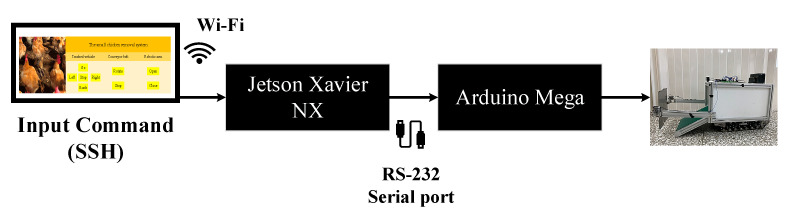
Signal transmission architecture.

Figure 3 is the signal transmission architecture. The evaluation board (EVB) used in the designed system includes the Jetson Xavier NX, produced by the NVIDIA company, and Arduino Mega, designed by the Arduino company. The Jetson Xavier NX, the main controller, is responsible for receiving, processing, and transmitting commands. The built-in Wi-Fi chip of the Jetson Xavier NX can communicate with a computer through the SSH (Secure Shell) protocol. The Jetson Xavier NX communicates with the Arduino Mega through a serial port. The Arduino Mega is responsible for the server-side execution of the overall software as well as receiving and executing commands. Its built-in serial port can receive commands with the Jetson Xavier NX through RS-232.Moreover, the chip can use a wireless LAN for command transmission and program execution. After Python has processed a command, it uses the built-in serial port in the Jetson Xavier NX to send ASCII characters.

### 3.3. Hardware Architecture, Circuit Design, and Actuation of the Designed System

**Figure 4:**
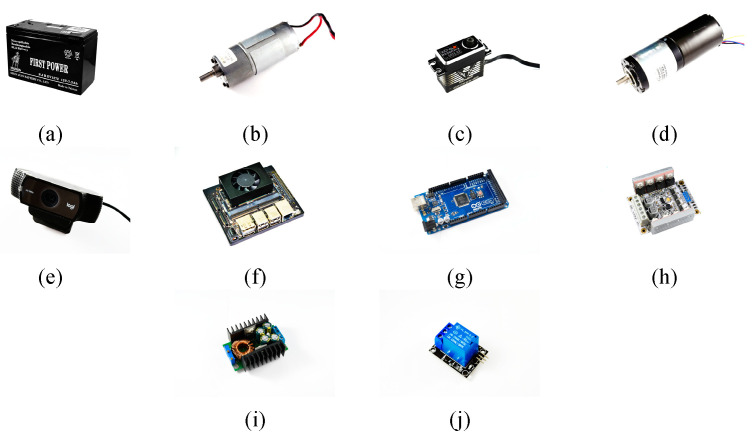
Components of the developed system: (**a**) battery, (**b**) DC brushed motor of the tracked vehicle, (**c**) servo motor of the robotic arms, (**d**) DC brushless motor of the conveyor belt, (**e**) visible light camera, (**f**) Jetson Xavier NX, (**g**) Arduino Mega, (**h**) DC motor shield, (**i**) buck converter, and (**j**) relay.

The materials used in the hardware design of the developed system, including the motors, EVB, and cameras, are displayed in Figure 4. When the motor is selected, the function of its actuation and the required characteristics, including the speed and torque, must be considered. In this study, three motors, namely a brushed DC motor (JGB37-555), servo motor (SB-2290SG), and brushless DC motor (CHP-42GP-4260), control the actions of different components. The brushed DC motor drives the walking of the tracked vehicle; the servo motor controls the robotic arm movement; and the DC brushless motor controls the rotation of the conveyor belt.

**Figure 5:**
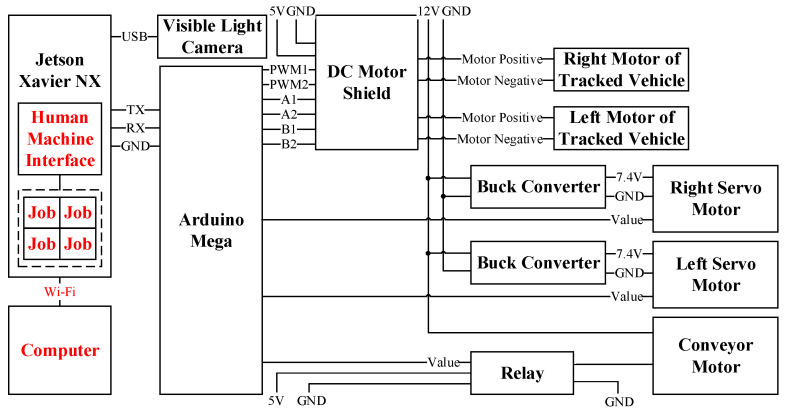
Hardware architecture of the designed system.

**Figure 6:**
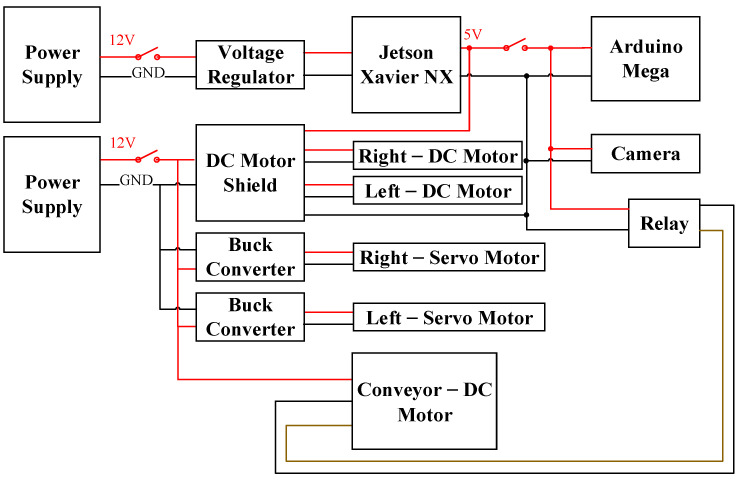
Circuit design of the developed system.

The EVB contains the Jetson Xavier NX; Arduino Mega; a DC motor shield, which controls the signal and pulse width modulation (PWM) of the DC motor; and a buck converter, which stabilizes and steps down the voltage to supply the voltage required by different components. The hardware architecture and circuit design of the developed system are illustrated in Figure 5 and Figure 6, respectively. Two 12-V batteries constitute the power supply of the entire system. One battery provides the power required by the motors, namely two brushless DC motors, two servo motors, and one DC brushed motor.

The brushless DC motors are controlled for PWM and for sensing the rotation of the DC motor shield. As the voltage required by the two servo motors is 7.4 V, the buck converter steps the voltage down from 12 to 7.4 V. The other supplies power to the Jetson Xavier NX. To improve the stability of the received voltage for the Jetson Xavier NX, a voltage regulator is connected between the power supply and the Jetson Xavier NX. The voltage required by other components of the EVB, including the Arduino Mega as well as the visible light camera and relay, is 5.0 V, which is supplied by the Jetson Xavier NX.

### 3.4. Behavior of the Removal System

The removal system is combined with the functions of the hardware components and the control of software programs, including the walking system, camera image return, object detection, and removable device operations to enable breeders to control the system using the remote control and automatic modes.

#### 3.4.1. Remote Control Mode

**Figure 7:**
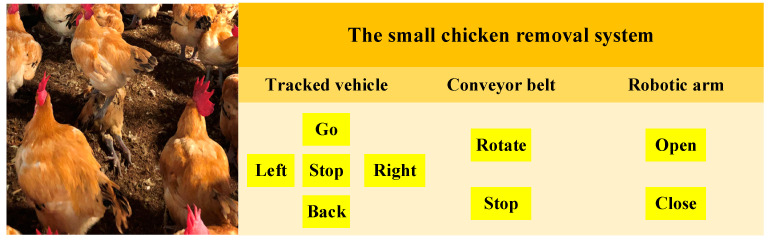
The human–machine interface of the designed system.

The human–machine interface of the designed system is shown in Figure 7. By using the designed human–machine interface for remote control, breeders can reduce the number of times they must enter a poultry house. This human–machine interface was developed using Tkinter, which is the standard Python interface in the Tk GUI toolkit [31]. The aforementioned interface allows the operator to perform tasks, such as controlling the walking system, controlling the conveyor belt, and opening and closing the robotic arm.

The orange square in Figure 7 indicates the title of the system; the dark yellow squares represent the labels, including the tracked vehicle, conveyor belt, and robotic arm; and the light yellow squares represent the buttons that the breeder can click to control the system. The developed system can also transmit images through the camera for monitoring the status of chickens in the poultry house in real time. Breeders can control the system according to the image information obtained from the poultry house and determine whether the chickens are in a good environment and healthy.

**Figure 8:**
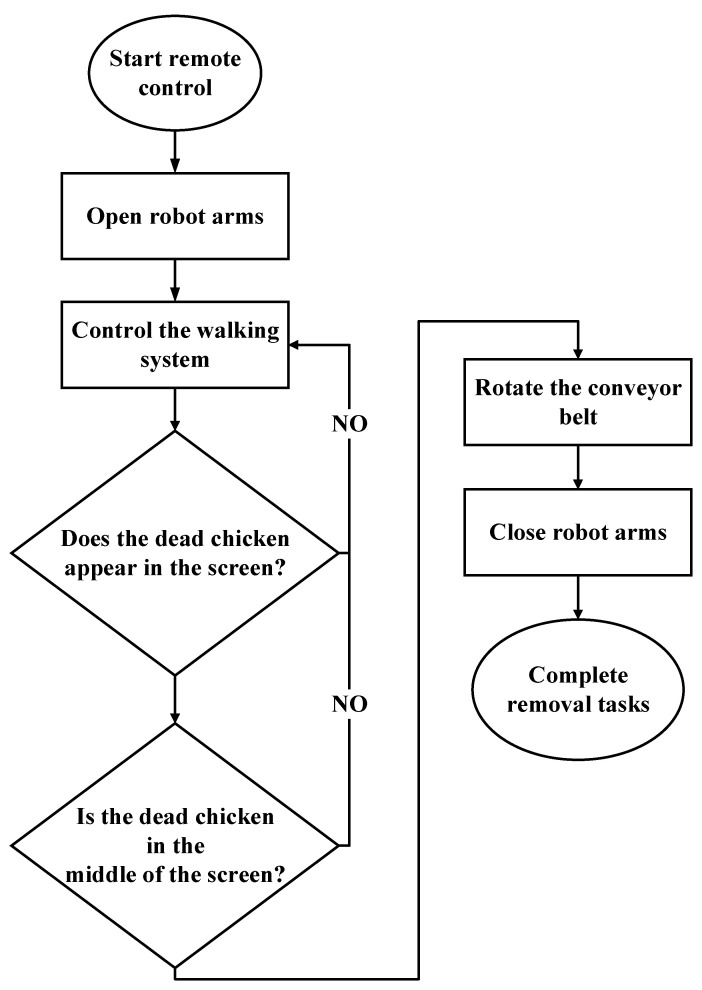
The remote control procedure for the developed system.

The remote control procedure for the developed system is displayed in Figure 8. The breeder controls the walking system to observe the chickens and find dead chickens. When a dead chicken appears on the screen, the breeder can adjust the position and distance between the system and the dead chicken and then start the conveyor belt and robotic arms to move the dead chicken into the storage cache.

#### 3.4.2. Automatic Mode

**Figure 9:**
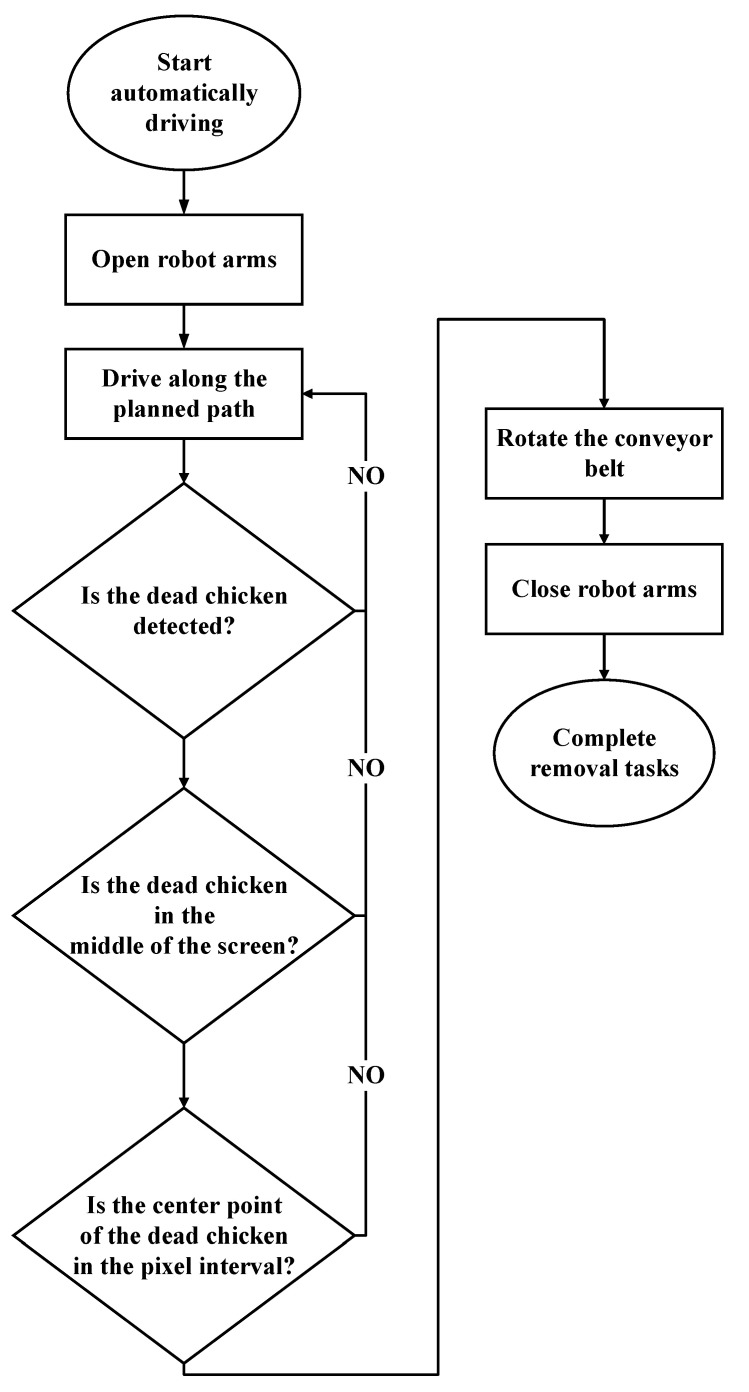
The functioning of the developed system in the automatic mode.

Figure 9 illustrates the functioning of the developed system in the automatic mode. First, the sweep-in device of the robot arms is opened such that dead chickens can be detected through deep learning. Next, the system moves along a straight path with a walking speed of 3.3 cm/s in the poultry house to search for dead chickens. When a dead chicken is identified by the system, the dead chicken must be in the middle of the screen. The obtained image has a resolution of 480 × 720 pixels. If the location of the dead chicken has *X*-axis values between 160 and 320, the center of the dead chicken should be set to *Y*-axis values of between 500 and 720 before the conveyor belt and robot arms can be started. The conveyor belt rotates, and the sweep-in device of the robotic arm subsequently closes. The dead chicken is then swept onto the conveyor belt and transported to the storage cache. Finally, the dead chicken is removed from the storage cache.

## 4. Object Detection

In the remote control mode, breeders can observe real-time images from the camera in the human–machine interface to identify dead chickens on the basis of their breeding experience. Alternatively, the system can automatically perform all tasks in the automatic mode. In the automatic mode, the deep learning method was adopted in the chicken removal system in order to reduce the time of breeders in the poultry house and the potential for virus spread.

Human contact with chickens can be decreased. Based on [32], Faster R-CNN, Tiny-YOLO v2, YOLO v3, SSD 300, EfficientDet-D0, and YOLO v4 have been proposed and tested in apple flower experiments. The mean average precision (mAP) and the detection speed of YOLO v4 are better than those of others. YOLO v4 satisfied the requirements of real-time monitoring. Thus, in our study, the designed system detects dead chickens using the YOLO v4 algorithm.

### 4.1. Methodology

#### 4.1.1. Dataset

This study used the images collected by a visible light camera and deep learning to detect dead chickens. The deep learning model was trained with an appropriate amount of image data, and the trained deep learning model was used to detect dead chickens. A large number of images of dead chickens were required for training to obtain a highly reliable trained model. The YOLO algorithm is based on a Convolutional Neural Network (CNN) [33]. YOLO v4 is more precise and feasibly used in the embedded system [34,35]. Table 2 shows the training layers of YOLO v4, YOLO v4-tiny, and YOLO v3 in this study. Table 3 shows the parameters of the YOLO v4 algorithm in this study.

For the dead chicken detection, a Logitech C922 Pro Stream camera was used to collect images of healthy chickens. Table 4 presents information on the collected data. Training data is used to train the model; validation data is used to select and modify the model, and test data is used to test whether the established model is accurate. A total of 150 images were collected. These images comprised 80 training images; 30 validation images; and 40 testing images, which consisted of 20 images each of dead and healthy chickens.

#### 4.1.2. Evaluation of the Model Performance

Many versions of the YOLO algorithm have been developed to enhance the system effectiveness [6,8,36]. Therefore, the effectiveness of the proposed method was verified by comparing it with two deep learning algorithms, namely YOLO v3 and Tiny YOLO v4. The classification results of the aforementioned algorithms were evaluated in terms of three indicators: precision, recall, and mAP. The confusion matrix of deep learning is an N × N matrix used for evaluating the performance of a classification model. The precision, which is expressed in Equation (1), is the rate of correct classification of dead chickens by the deep learning algorithm.

(1) $$\mathrm{Precision}=\frac{TP}{TP+FP}$$

where *TP* and *FP* are the numbers of true positives and false positives, respectively. High precision means that the system achieves superior identification results for the detection of dead chickens. The accuracy is expressed as follows:

(2) $$\mathrm{Accuracy}=\frac{TP+TN}{TP+TN+FP+FN}$$

where *TN* is the number of true negatives and *FN* is the number of false negatives.

Another indicator of algorithm performance is the recall, which is expressed as follows:

(3) $$\mathrm{Recall}=\frac{TP}{TP+FN}.$$

The recall indicates how many dead chickens are successfully detected among all the dead chickens. Therefore, the higher the recall, the better the identification results. The intersection over union (IOU) is a suitable metric for measuring the overlap between two bounding boxes. This parameter is defined as follows:

(4) $$\mathrm{Intersection}\;\mathrm{Over}\;\mathrm{Union}=\frac{\left|R\cap^{}\hat{R}\right|}{\left|R\cup^{}\hat{R}\right|}$$

where *R* is the predicted bounding box and $\hat{R}\;$ is the ground-truth bounding box. An IOU value of ≥0.5 indicates a true positive, whereas an IOU value of <0.5 indicates a false positive. The mAP is defined as follows:

(5) $$\mathrm{mAP}=\frac{\sum_{C=1}^{C}AP\left(C\right)}{C}$$

where C is the number of detection categories. As only chickens were detected in this study, the value of C is 1 in this study. The higher the mAP, the better the performance of the detection system for the identification of dead chickens. Table 5 indicates that the YOLO v4 algorithm had an mAP value of 100% for dead chicken detection, 23.86% and 25.27% higher than those of the YOLO v3 and Tiny YOLO v4 algorithms, respectively, when the IOU value was 0.5.

Table 6 shows the performances of the compared algorithms when the IOU value was 0.75. When the IOU value was 0.75, the YOLO v4 algorithm had an mAP value of 82.39%, which is 64.52% and 60.21% higher than those of the YOLO v3 and Tiny YOLO v4 algorithms, respectively. The YOLO v4 algorithm outperformed the other algorithms in the real-time and accurate detection of dead chickens. Thus, the YOLO v4 algorithm was integrated with the removal system for the automatic removal of dead chickens.

### 4.2. Results and Analysis

Identifying dead chickens through deep learning can reduce the labor requirements and increase the intelligent functions of the developed system. Therefore, we used the YOLO v4 algorithm to detect dead chickens. When the IOU value was set to 0.5, the precision, accuracy, and recall were 95.24%, 97.5%, and 100%, respectively (Table 7). The aforementioned values are higher than 90%, which indicates that the trained model had a high degree of reliability.

**Figure 10:**
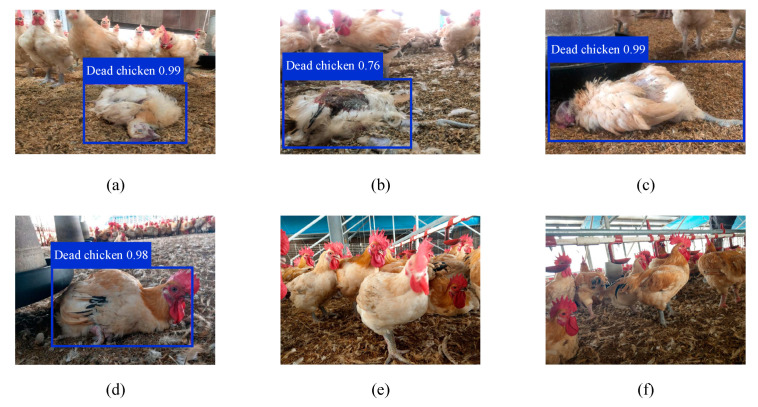
Identification results obtained with the developed system: (**a**–**c**) successful identification of dead chickens, (**d**) healthy chicken identified as a dead chicken, and (**e**,**f**) healthy chickens not identified.

If the developed system identifies a dead chicken in an input image, the target of the dead chicken is framed and marked with the probability of a dead chicken being present, as illustrated in Figure 10. When the system does not identify a sick or dead chicken, no label is generated. For the data in Figure 10a–c, the probabilities of the system successfully identifying dead chickens were 99%, 76%, and 99%, respectively. The probability obtained for the data in Figure 10b was lower than those for data in Figure 10a,c because distinguishing the head and feet of the chicken was difficult in the case of Figure 10b. Figure 10d illustrates an incorrect classification of healthy chickens as being dead. The reason for this error was that the morphologies of some healthy chickens were similar to those of dead chickens, which caused errors in the deep learning recognition. Figure 10e,f illustrates the nonidentification of healthy chickens by the developed system.

Two reasons exist for the identification errors and decreased probability. The first reason is related to the incomplete shape of the dead chickens. If the head and feet of the dead chickens are not visible, the probability of the image classification is affected. The second reason is as follows: When a healthy chicken is sitting or lying down, identification errors occur because the morphology is similar to that of a dead chicken. If the training dataset of the model is enlarged, the identification error can be effectively reduced to improve the precision.

## 5. Evaluation of the Chicken Removal Performance

The developed system was used in a commercial Taiwanese poultry house to test the durability of the walking and removal systems, the removal performance, and the performance of the identification system.

### 5.1. Experimental Environment

**Figure 11:**
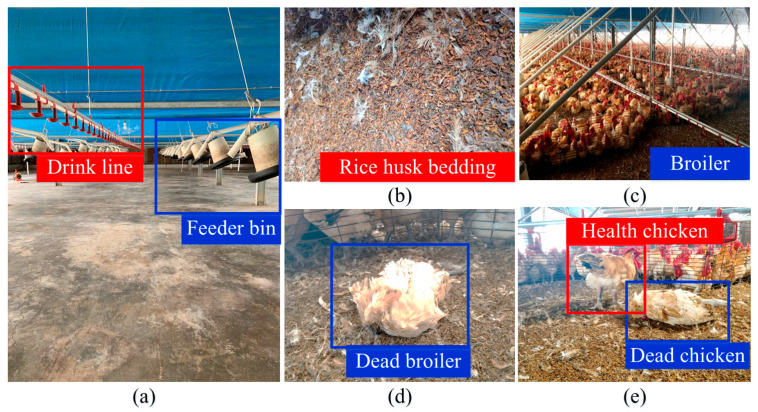
Experimental environments in which the developed system was used: (**a**) poultry house environment, (**b**) rice husk bedding, (**c**) experimental objects (broilers), (**d**) dead broiler, and (**e**) healthy and dead chickens.

**Figure 12:**
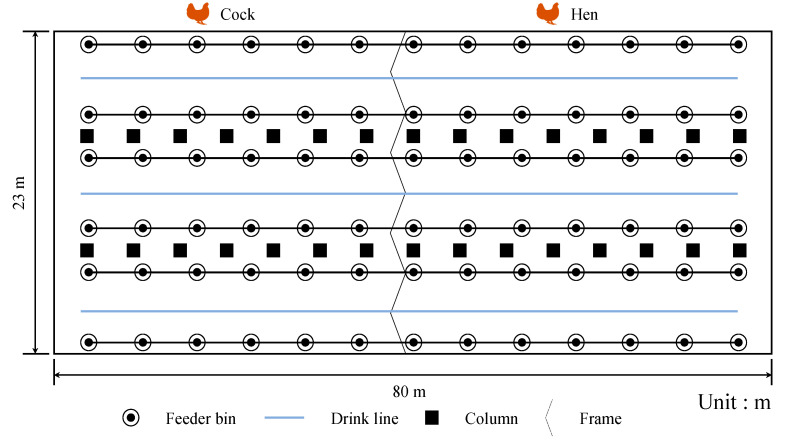
A schematic of the test environment. Black circles indicate feeder bins. Blue lines indicate drink lines. Black blocks indicate columns. Cocks and hens were separated by frames. The test environment had a length of 80 m in the X-direction and 23 m in the Y-direction.

Figure 11 illustrates experimental environments where the developed system was used. Figure 11a is the poultry house environment. Figure 11b is rice husk bedding. Figure 11c is the experimental objects—broiler chickens. Figure 11d is a dead broiler, and Figure 11e includes healthy and dead chickens. Before the developed system was used in the commercial poultry house, tests were conducted to verify that it could operate in a poultry house and remove dead chickens. The poultry house is a closed house with dimensions of approximately 80 × 23 × 4.5 m^3^. The top view of the commercial poultry house is displayed in Figure 12. The house contains feeder bins, drink lines, columns, and frames separating cocks and hens. Some information on the poultry house is listed as follows:

▪ The broilers are fed through floor feeding.
▪ The poultry house structure has a single layer.
▪ The feeding period is 3 months.
▪ The number of the broilers is approximately 18,000.
▪ The feeding density is 9.8/m^2^.

According to poultry pathology, a daily loss of less than 1/2000 chickens is reasonable. However, the breeder must be vigilant when the number of dead chickens exceeds 1/1000. However, the number of unhealthy chickens cannot be accurately determined, and the breeding rates of chickens at different stages are not the same. Only the number of dead chickens can be estimated.

### 5.2. Walking and Removal Systems

Walking speed and stability are the most important factors in the test of the walking system because the litter that covers the floor of the poultry house affects these factors the most. The wheels of the tracked vehicle can become easily stuck on the poultry house floor, making smooth walking impossible for the vehicle. Therefore, it is necessary to test the motor performance to be sufficient to load the weight of the whole system and to have sufficient friction with the litter to drive the operation of the system. The PWM of the tracked vehicle motor determines the motor speed, and the PWM value is between 0 and 255. The higher the PWM value, the lower the motor torque.

After simulations for the floor of the poultry house were performed, the most appropriate PWM value was determined to be 180 to ensure sufficient friction for the tracked vehicle to walk stably on the litter. After experiments in the simulated poultry house environment and adjustment of the motor parameters, the walking system was tested in the real poultry house with a walking speed of 3.3 cm/s on the litter. In addition to avoiding large disturbances to the chickens, a low walking speed can prevent dust from being raised, which would affect the poultry house environment.

The removal system tests were mainly conducted to test the performance of the robotic arms, conveyor belt, and storage cache. The weight of an adult chicken is approximately 2–3 kg; therefore, whether the torque of the sweep-in device motor for the robotic arms generates sufficient force to sweep dead chickens must be determined. A servo motor with a torque of 50 kg-cm under an input voltage of 7.4 V was used to drive the rotation of the stainless steel plate used as the sweep-in device. The length of this plate and the aluminum extrusion of the sweep-in end was approximately 20 cm. The force on one side was approximately 2.5 kg; thus, the total force was approximately 5 kg, which was sufficient for sweeping dead chickens.

The conveyor belt was driven by a low-speed motor such that the torque could be increased. To increase the friction between the chickens and the conveyor belt, a rough and grooved belt was selected. Dead chickens were transported form the head side of the conveyor to the storage cache in 20 s. When the first dead chicken was transported to the storage cache, the next one was pushed by the friction of the conveyor belt. Due to the friction of the conveyor belt and the space of the storage cache, two chickens could be removed in a single operation. The functions of the walking and removal systems were integrated to remove dead chickens from the Taiwanese poultry house.

**Figure 13:**
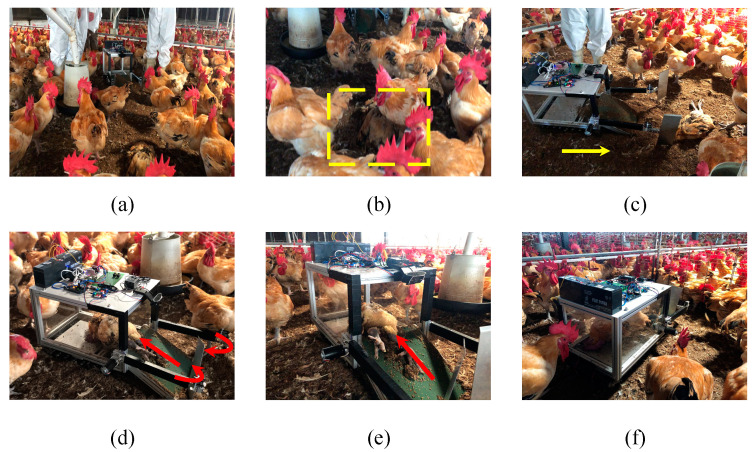
(**a**) Process of the developed system, (**b**) the identification of dead chickens, (**c**) inputting the command of the tracked vehicle, (**d**) inputting the commands of the conveyor belt and robot arms, (**e**) the movement of a dead chicken to the cache storage, and (**f**) the complete removal task.

When the system was approximately 1-m away from a dead chicken, the walking system required 33 s to reach the point where the dead chickens could be removed and approximately 20 s to remove and transport the chicken to the storage cache. Considering the delay in signal transmission, the system could remove one dead chicken in approximately 1 min. As displayed in Figure 13, the designed system successfully identified dead chickens in the commercial poultry house and moved them to the storage cache.

## 6. Conclusions

In this study, we designed a small system for removing dead chickens from the floors of Taiwanese poultry houses. This system does not cause any stress to healthy chickens. Instead, the removal system can promote their activities. The removal method of the designed system involves sweeping dead chickens into a storage cache by using fixed aluminum extrusions and rotatable stainless steel plates. The designed system has dimensions of approximately 1.038 × 0.36 × 0.5 m^3^.

The designed system detects dead chickens through deep learning based on the YOLO v4 algorithm. The precision of the system was approximately 95.24%, the accuracy was 97.5%, and the recall was 100%. The walking speed of the chicken removal system was 3.3 cm/s in a real poultry house, and it could remove two dead chickens in a single operation. When the designed system was approximately 1-m away from a dead chicken, it took approximately 1 min to completely remove it, including the walking time, removal time, and signal transmission delay.

The designed system had two modes for removing dead chickens: remote control and automatic. In the remote control mode, images captured by the system camera were transmitted to and displayed on a human–machine interface for the remote monitoring of chickens in poultry houses. In the automatic mode, deep learning is integrated with a self-propelled vehicle to identify dead chickens using the YOLO v4 algorithm. Thus, the developed system can not only quickly and effectively remove dead chickens, which are sources of pollution, but can also quickly restore the breeding environment to avoid the spread of pathogens that can affect the growth of healthy chickens.

Only one to two people are required to complete the operations of the developed system. Therefore, the labor cost required by the breeder and the amount of human contact with chickens can be reduced. Consequently, the possibility of artificially introducing germs to the poultry house can also be reduced. Furthermore, the removal system can reduce the possibility of secondary pollution or multiple infections and, thus, improve biological safety. As the recognized shapes of the dead chickens are incomplete and the morphology is similar to a dead chicken if a healthy chicken is sitting or lying down, the probability of the image classification is affected. Therefore, in increasing the training datasets of the model, the identification errors can be effectively reduced to improve the precision and the accuracy.

The designed system is economically efficient. It can not only improve the problem of labor shortages and reduce labor costs but can also improve the breeding environment to increase the overall breeding rates of chickens and the economic efficiency of breeding. The use of robots and sensors in Taiwanese poultry houses enhances the application of artificial intelligence and automation in these houses and provides various benefits to the Taiwanese poultry industry.

## Figures and Tables

**Table 1:** Components and systems of the designed chicken removal system.

System	Component	Model
Walking system	Tracked vehicle DC motor	WT-500 (L: 500 mm; W: 300 mm; H: 120 mm) JGB 37-555 (DC12 V, 22 RPM)
Robotic arm	Servo motor Aluminum extrusionStainless steel	SB-2290SG (DC 7.4 V) L:350 mm L: 300 mm; W: 110 mm
Conveyor belt	DC motor Belt Aluminum extrusion	CHP-42GP-4260 (DC12 V 1:92) PVC (L: 720 mm; W: 259 mm) L: 260 mm
Storage cache	Aluminum extrusion Transparent acrylic sheet	L: 450 mm; W: 300 mm; H: 250 mm

**Table 2:** The training layers of YOLO v4, YOLO v4-tiny, and YOLO v3.

Layers	YOLO v4	YOLO v4-Tiny	YOLO v3
convolutional	110	21	75
route	21	11	4
shortcut	23	0	23
maxpool	3	3	0
upsample	2	1	2

**Table 3:** The parameters of the YOLO v4 algorithm in this study.

Parameters	
batch	64
subdivisions	64
width height	800 800
max_batches	10,000
classes	1
filters	18

**Table 4:** Information on the collected data.

Datasets	Images
Training Data	80
Validation Data	30
Test Data (Dead chicken) Test Data (Health chicken)	20 20

**Table 5:** Performance of the compared algorithms when the IOU value was 0.5.

IOU = 0.5	mAP (%)
YOLO v3	76.14
Tiny-YOLO v4	74.73
YOLO v4	100

**Table 6:** Performance of the compared algorithms when the IOU value was 0.75.

IOU = 0.75	mAP (%)
YOLO v3	17.87
Tiny-YOLO v4	22.18
YOLO v4	82.39

**Table 7:** Identification of the results obtained with the developed system.

IOU = 0.5		Predicted Label	
		Positive	Negative
True Label	Positive	(True Positive) TP = 20	(False Negative) FN = 0
	Negative	(False Positive) FP = 1	(True Negative) TN = 19
Precision		95.24%	
Accuracy		97.5%	
Recall		100%	

## Data Availability

Not applicable.
